# Capture heats up sharks

**DOI:** 10.1093/conphys/coac065

**Published:** 2022-09-28

**Authors:** Lucy Harding, Austin Gallagher, Andrew Jackson, Jenny Bortoluzzi, Haley R Dolton, Brendan Shea, Luke Harman, David Edwards, Nicholas Payne

**Affiliations:** Department of Zoology, Trinity College Dublin, D02 PN40, Ireland; Beneath the Waves, PO BOX 126, Herndon, VA 20172, USA; Department of Zoology, Trinity College Dublin, D02 PN40, Ireland; Department of Zoology, Trinity College Dublin, D02 PN40, Ireland; Department of Zoology, Trinity College Dublin, D02 PN40, Ireland; Beneath the Waves, PO BOX 126, Herndon, VA 20172, USA; School of Biological, Earth and Environmental Sciences, University College Cork, Distillery Fields, North Mall, Cork, T23 N73K, Ireland; West Cork Charters, Shannonvale, Clonakilty, Co. Cork, , P85 FV00, Ireland; Department of Zoology, Trinity College Dublin, D02 PN40, Ireland

**Keywords:** thermal ecology, shark, physiology, catch-and-release, capture, body temperature

## Abstract

Catch-and-release fishing is an important component of ecotourism industries and scientific research worldwide, but its total impact on animal physiology, health and survival is understudied for many species of fishes, particularly sharks. We combined biologging and blood chemistry to explore how this fisheries interaction influenced the physiology of two widely distributed, highly migratory shark species: the blue shark (*Prionace glauca*) and the tiger shark (*Galeocerdo cuvier*). Nineteen sharks were caught by drum line or rod-and-reel angling; subcutaneous body temperature measurements were taken immediately upon capture, with six individuals also providing subsequent subcutaneous body temperature measurements via biologging as they swam freely for several hours post-release. We found that short-term capture caused shark body temperature to increase significantly and rapidly, with increases of 0.6°C–2.7°C for blue sharks (mean, 1.2 ± 0.6°C) and 0.5°C–0.9°C for tiger sharks (mean, 0.7 ± 0.2°C) and with capture-induced heating rates of blue sharks averaging 0.3°C min^−1^ but as high as 0.8°C min^−1^. Blue shark body temperature was even higher deeper into the white muscle. These heating rates were three to eight times faster than maximum rates encountered by our biologging sharks swimming through thermally stratified waters and faster than most acute heating experiments conducted with ectotherms in laboratory experiments. Biologging data showed that body temperatures underwent gradual decline after release, returning to match water temperatures 10–40 mins post-release. Blood biochemistry showed variable lactate/glucose levels following capture; however, these concentrations were not correlated with the magnitude of body temperature increase, nor with body size or hooking time. These perturbations of the natural state could have immediate and longer-term effects on the welfare and ecology of sharks caught in catch-and-release fisheries and we encourage further study of the broader implications of this reported phenomenon.

## Introduction

Catch-and-release fishing is a popular practice in recreational fisheries (Cooke and Schramm, 2007; Danylchuk *et al.,* 2014; Brownscombe *et al.,* 2017) and is often used in scientific research (Wosnick *et al.,* 2018)—in particular, tagging studies (Watanabe *et al.,* 2015; Royer *et al.,* 2020; Harding *et al.,* 2021). Catch-and-release can also occur on commercial fishing vessels where it has been estimated that ~9.1 million tonnes of non-target fish per year globally are caught and released in commercial fisheries (Sepulveda *et al.,* 2019; Gilman *et al.,* 2020). Numerous studies have investigated the physiological and ecological impacts of catch-and-release fishing on teleosts and elasmobranchs (Cooke and Schramm, 2007; Cooke *et al.,* 2013; Gale *et al.,* 2013; Gallagher *et al.,* 2014; Schlenker *et al.,* 2016; Jerome *et al.,* 2017; Gallagher *et al.,* 2019; Sepulveda *et al.,* 2019; Mohan *et al.,* 2020). Catch-and-release fishing is considered a multi-stressor interaction for individuals (Gale *et al.,* 2013), with the negative impacts broadly categorised as lethal or sub-lethal, with potential sub-lethal impacts including altered blood chemistry (Gallagher *et al.,* 2014; Dapp *et al.,* 2016), behavioural impairments (Raoult *et al.,* 2019), reduced growth and reproductive rates and increased disease vulnerability (Gale *et al.,* 2013). Fish can experience oxygen deprivation (Schwieterman *et al.,* 2021), handling-related injuries (Cooke *et al.,* 2013; French *et al.,* 2015) and heat stress (Wosnick *et al.,* 2018, 2019).

Many studies investigating the impacts of catch-and-release fishing have highlighted the potential importance of temperature in these interactions (Meka and McCormick, 2005; Gale *et al.,* 2013; Schlenker *et al.,* 2016; Bouyoucos *et al.,* 2018; Schwieterman *et al.,* 2021), whereby water temperature (T_a_) can have a significant effect on post-release survival and a variety of sub-lethal impacts (Gale *et al.,* 2013). Furthermore, body temperature (T_b_) is considered an important parameter when assessing an individual’s response to stress, their metabolic rate and their energy budgets; all of which may be affected by catch-and-release interactions. However, few studies have measured fish body temperatures during catch-and-release. Measurements of muscle temperature are especially rare, and while two recent studies have inferred surface temperature of sharks’ post-capture using thermal imaging of shark skin (Wosnick *et al.,* 2018, 2019), reflectance issues and effects of varying sun and water exposure likely complicate estimates of true operative body temperatures. Sharks are relatively large-bodied fishes with low heat-transfer coefficients (Nakamura *et al.,* 2020) so capture-induced heating could be a particularly relevant issue for them.

When hooked, sharks often exhibit intensive swimming acceleration (Gallagher *et al.,* 2017) and periods of exhaustive exercise (i.e. anaerobic exercise) (Schwieterman *et al.,* 2021), as they attempt to escape. Capture-related exercise and the resulting physiological stress can elevate the metabolic rate (Kieffer, 2000; Mohan *et al.,* 2020) resulting in, among other things, the generation of heat. Furthermore, fish are known to dissipate heat across the gill surface during respiration (Stevens and Fry, 1974; Stevens, 2011; Nakamura *et al.,* 2020) and so during the period of fighting on the hook, these animals may be unable to swim freely, which could provide a mechanism for reduction in heat loss. This excess heat may manifest as an elevation of body temperature in these animals and thus, this study aims to investigate this relationship and determine the extent to which sharks heat up when captured.

In addition to altered thermal dynamics, hooked fish may undergo altered blood biochemistry as a result of the event (Gallagher *et al.,* 2014; Dapp *et al.,* 2016; Mohan *et al.,* 2020; Schwieterman *et al.,* 2021). Capture can often result in hyperkalemia, metabolic and respiratory acidosis (Schwieterman *et al.,* 2021) and declines in blood oxygen concentration, as a result of the often exhaustive exercise and apneic asphyxia associated with capture (Schwieterman *et al.,* 2021). Lactate and glucose are two metabolites widely examined in studies investigating stress in sharks (Cliff and Thurman, 1984; Hoffmayer and Parsons, 2001; Mandelman and Skomal, 2008; Gallagher *et al.,* 2014, 2017). Lactate is a metabolite that is produced anaerobically in the white muscle during exhaustive exercise (Moyes *et al.,* 2006). Glucose is measured as a proxy for the glucocorticoid hormone stress response whereby hepatic glycogen is converted to glucose during gluconeogenesis and released to fuel muscle tissues (Hoffmayer and Parsons, 2001; Prohaska *et al.,* 2021). Body temperature and lactate and glucose are both key elements of metabolic processes. Therefore, by combining our investigations into the body temperature measurements with the blood biochemistry data, we can explore how the two might be related during catch-and-release events.

## Materials and Methods

### Data collection

To properly evaluate body temperature dynamics in captured marine predatory fishes, we incorporated all phases of the capture and release interaction, from moment of hooking to several hours post-release.

#### Biologging

We used biologging technology to collect fine-scale, physiological measurements from individuals free-swimming in the wild. Fishing was conducted across three locations: the Bahamas in May 2019, Cape Cod, USA in September 2019 and Co. Cork, Ireland in July–October 2021. We captured tiger sharks by drum lines and blue sharks by rod-and-reel angling. Tiger sharks were secured alongside the boat (remaining submerged during the procedure) and blue sharks were brought on deck with a deck hose placed in the mouth to constantly irrigate the gills with water taken from the ocean surface. Biologging packages were fitted to the first dorsal fin of four tiger sharks (*Galeocerdo cuvier*) and two blue sharks (*Prionace glauca*) (Supplementary Table S1), which were then immediately released. A plastic cable was passed through two 1-cm incisions made in the dorsal fin, which was connected to a dissolvable time-release mechanism that secured the package to the fin. Biologging packages included a mixture of loggers such as accelerometers (recording tri-axial acceleration at 25 Hz and depth at 1 Hz; Techno-Smart AGM-1; 67 × 42 × 19 mm), animal-borne digital cameras (recording at 30 fps; Little Leonardo DVL400M065; 61 × 21 × 15 mm; 29 g in air; 4 x red LED lights) and temperature loggers (recording ambient and body temperature at 1 Hz; Lotek LAT 1810; 11 × 38 mm, 7.6 g in air; Wildlife Computers Mk9; 72 × 17 × 17 mm, 34 g in air). Body temperature was measured via a sensor stalk inserted 4–8 cm into the dorsal musculature of the shark, adjacent to the dorsal fin. To enable retrieval, tag packages also included a VHF transmitter (Advanced Telemetry Systems, MM100) and satellite position-only tag (Wildlife Computers Model 258; ARGOS enabled). Once the time-release mechanism dissolved, the package detached from the fish and floated to the surface, as they were constructed of a positively buoyant material (Diab Syntactic © non-compressible foam). Packages were then located using the ARGOS system and a VHF receiver and retrieved from the ocean surface by boat. A total of four tiger sharks (ranging 155.7–206 kg body mass) and two blue sharks (ranging 25.4–30.7 kg body mass) were tagged.

#### Additional body temperature measurements of blue sharks

In addition to the biologging work, we collected body temperature measurements from 13 additional blue sharks, from the moment they were landed on deck, until the moment before release (Supplementary Table S1). Temperature probes were fitted to the sharks using the same method as the biologging. The temperature probe was left in place while a work-up was completed on the shark (e.g. recording biometrics, collecting blood samples, etc). Once the work-up was completed, the temperature probe was removed from the muscle and the shark was released. Water temperature was recorded by subsequently placing the temperature logger in the surface water for approx. 2 mins. Additionally, secondary body temperature measurements were taken from 4 of these 12 sharks (i.e. BS32–BS35) to generate a thermal profile of the shark’s musculature. To do this, the temperature probe was inserted at a depth of 2 cm into the dorsomusculature, ~20 cm more anterior and closer to the gills than the first probe, left for 1–2 mins, pushed a further 2 cm into the dorsomusculature (to measure body temperature at 4 cm depth) and left for the remaining period of time that the shark was held on deck. The probe was then removed from the shark prior to release.

#### Caudal peduncle blood sampling

For 14 blue sharks, two blood samples were collected from the caudal peduncle using an 18-gauge needle: one immediately upon capture and one the moment before release (~10 mins later), corresponding with the timing of insertion and removal of the temperature stalk. The time of collection was recorded, and the samples were processed for lactate and glucose immediately after collection on deck, using a HaB direct Lactate Pro™ 2 lactate meter (HaB Direct, 2021) and Accu-Chek Performa blood glucose meter (Accu-Chek, 2021), respectively. A number of lactate/glucose readings could not be recorded due to logistical constraints and/or and user error.

#### Ethics

All works carried out in Ireland were conducted under The Health Products Regulatory Authority (HPRA) Project Authorisation (AE19136/P127). All works carried out outside of Ireland were conducted under local licensing, obtained by Beneath the Waves.

### Data analyses

Data handling and statistical analyses were carried out in R Version 4.0.3 (R Core Team, 2020) and IGOR Pro 8 (WaveMetrics, 2020) with Ethographer package (Sakamoto *et al.,* 2009). Time-series analyses were conducted on all biologging data with a focus on the body temperature, water temperature and depth data. A 500-point moving average smoother was applied to the temperature and depth data when plotting the time series.

Upon capture—meaning the moment individuals were landed on deck or secured alongside the boat—the metric ‘∆T’ was calculated as the difference between body temperature T_b_ and water temperature T_a_. The total ‘hooking time’ for each blue shark was calculated (i.e. the time from when the shark was hooked, to when it was brought on deck). Hooking time could not be calculated for the tiger sharks as they were caught using drum lines. Body mass was estimated for each individual using the length–weight relationship *W = aL^b^* where *W* is weight/body mass (kg), *L* is fork length (cm) and *a* and *b* are published, species-specific coefficients with *a* = 3.184 × 10^−6^ and *b* = 3.1313 for blue sharks and *a* = 2.528 × 10^−6^ and *b* = 3.2603 for tiger sharks (Kohler *et al.,* 1995).

Multiple Gaussian generalised linear models were conducted to investigate potential relationships between ΔT and hooking time, body mass, lactate concentration and glucose concentration (Supplementary Table S2). Initial and subsequent lactate and glucose concentrations were compared in order to investigate potential lags in stress biomarker elevations following capture.

## Results

A total of 19 sharks were caught: 4 tiger sharks and 15 blue sharks. Biologging data were collected from 6 of these individuals (4 tiger sharks and 2 blue sharks), and blood samples were taken from 14 blue sharks and no tiger sharks (Supplementary Table S1). Subsequently, one individual (tiger shark, T2) was excluded from all analyses as it was found to exhibit faulty biologging data.

**Figure 1:**
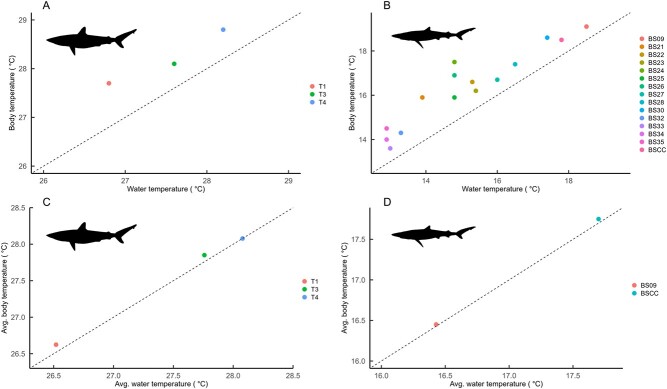
Body temperature elevation of tiger sharks and blue sharks recorded immediately upon capture (panels A and B) and average elevation of body temperature, during a 30-minute period under natural conditions several hours after release (panels C and D). Dashed black lines indicate a 1:1 identity line. (Blue shark image created by Ignacio Contreras and reproduced under the Creative Commons Attribution 3.0 Unported license: https://creativecommons.org/licenses/by/3.0/legalcode).

At the moment of capture, all sharks across both species exhibited elevated T_b_ relative to T_a_ (Fig. 1A and B), with elevations ranging from 0.6°C to 2.7°C for blue sharks (mean ± SD = 1.2 ± 0.6°C) and 0.5°C to 0.9°C for tiger sharks (mean ± SD = 0.7 ± 0.2°C) (Supplementary Table S1).

**Figure 2:**
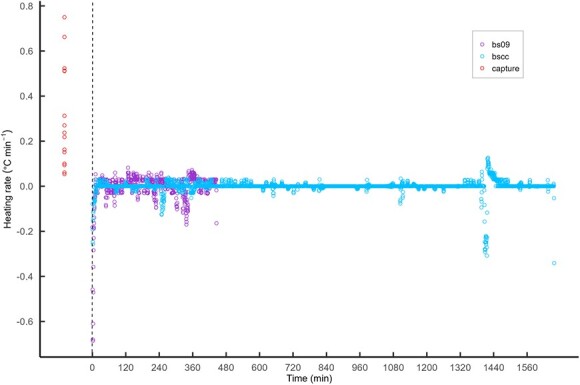
Average heating rate (°C min^−1^) for each blue shark during capture period (red circles; *n* = 15) and average heating rate every 30 sec for two biologged blue sharks (BSCC and BS09) during post-release phase (*n* = 4205). Capture datapoints (red circles) are placed arbitrarily to left of x-axis to illustrate their collection before the beginning of the time series post-release.

This elevation in temperature occurred over short periods of time (i.e. hooking time), which ranged from 1.4 to 13 mins for blue sharks (*n* = 15), indicating that warming occurred at an estimated rate of 0.1–0.8°C min^−1^ (mean ± SD = 0.3 ± 0.23°C min^−1^). These warming rates were unusually high when compared with the post-release data taken from biologged blue sharks BS09 and BSCC (Fig. 2). Free-swimming blue sharks rarely exhibited heating rates greater than 0.1°C min^−1^ (Fig. 2).

**Figure 3:**
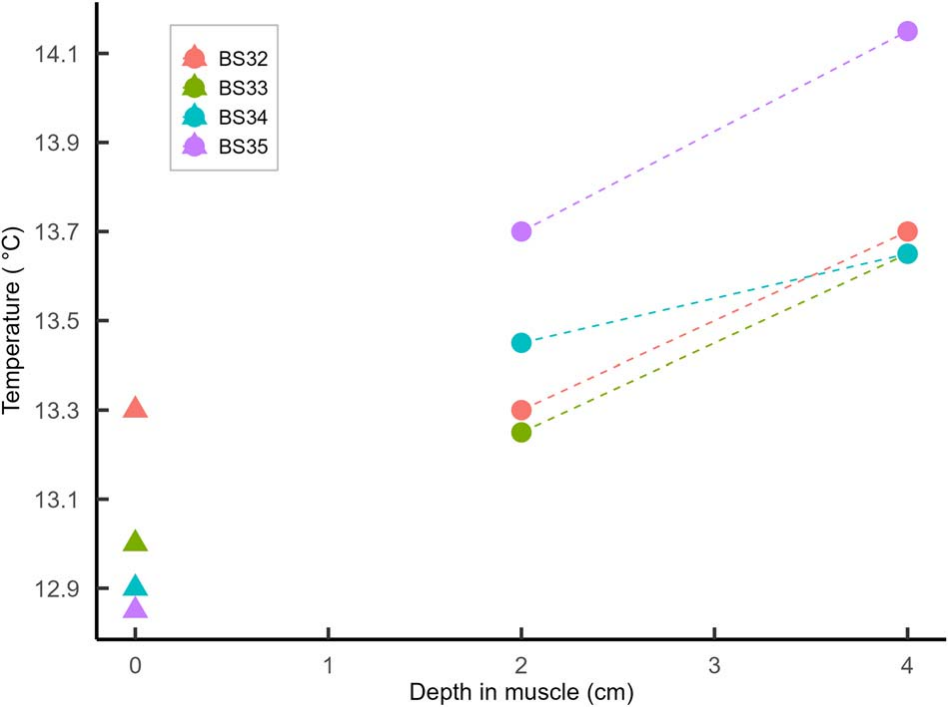
Water temperature measurements (triangles) and body temperature measurements (circles) taken at 2 and 4 cm penetration into the dorsal musculature of four blue sharks.

**Figure 4:**
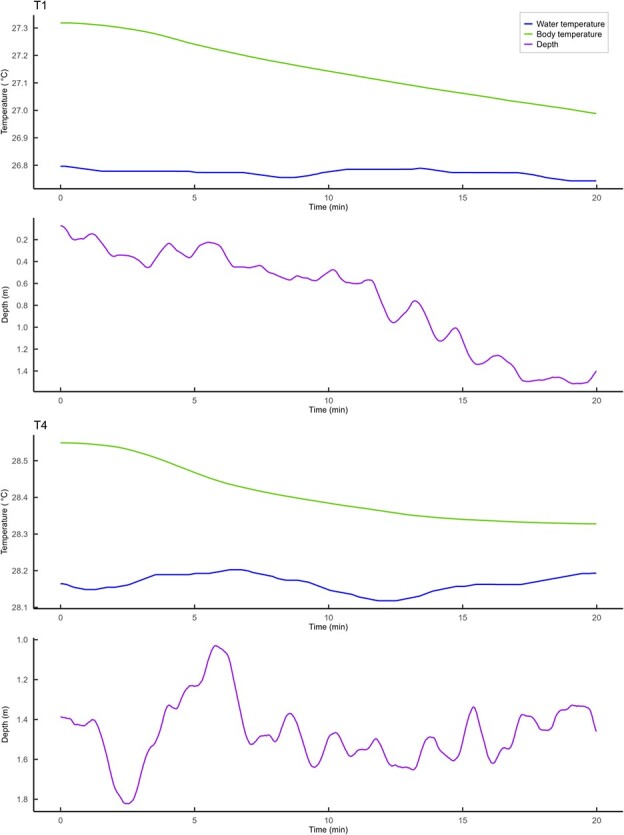
Body temperature, water temperature and depth data for tiger sharks T1 (top panel) and T4 (bottom panel) from moment of release. A 500-point moving average smoother was applied to all data.

The additional body temperature measurements taken from individuals BS32–BS35 revealed a further increase in body temperature, the deeper the temperature probe was inserted, with the difference between readings at 2 cm penetration and 4 cm penetration ranging from 0.20°C to 0.45°C (Fig. 3). The relationship between water temperature and deep muscle temperature was approximately linear despite a somewhat variable relationship between water temperature and shallow muscle temperature (Fig. 3), perhaps representing an uncertainty as to what depth and therefore temperature these sharks were initially hooked at. For all sharks equipped with biologging packages and subsequently released, following the initial elevation of T_b_ upon capture, T_b_ underwent a gradual decline, over the first 30 mins for tiger sharks and 15 mins for blue sharks, before returning to match water temperature. During this period of body temperature decline, there was no corresponding decline in water temperature or depth (Fig. 4). Several hours after release, T_b_ essentially matched T_a_ during extended periods (Fig. 1C and D).

**Figure 5:**
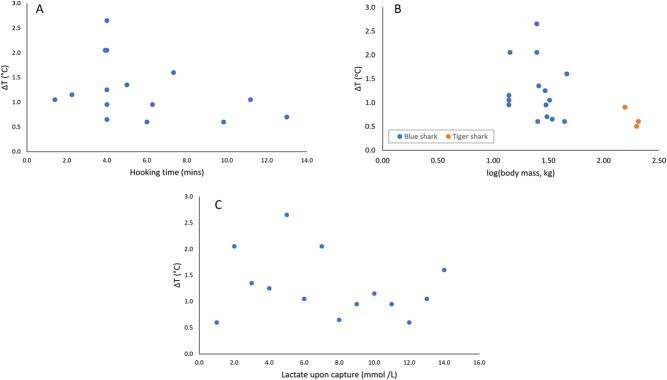
(A–C): (A) Temperature differential (ΔT) against hooking time for blue sharks with no significant relationship. (B) Temperature differential (ΔT) against log (body mass) with no significant relationship. (C) Temperature differential (ΔT) against lactate upon capture for blue sharks with no significant relationship.

Regression analyses showed hooking time has no significant influence on ΔT (Fig. 5A; *P* = 0.44); log-transformed body mass has no significant influence on ΔT (Fig. 5B; *P* = 0.12); lactate concentration has no significant influence on ΔT (Fig. 5C; *P* = 0.45); and glucose concentration has no significant influence on ΔT (*P* = 0.34). Multiple linear regressions with ΔT as the response variable and hooking time, body mass, lactate concentration and glucose concentration as predictor variables showed no significant relationships (see Supplementary Table S2, for more details).

Blue shark blood analysis showed lactate levels upon capture ranged from 1.2 to 9.0 mmol l^−1^ (mean = 3.1 mmol l^−1^, *n* = 13). Following time on deck lactate levels ranged from 2.9 to 9.0 mmol l^−1^ (mean = 5.4 mmol l^−1^, *n* = 11). Furthermore, 9 out of 11 blue sharks tested twice showed increased lactate levels following time on deck (mean increase ± SD = 2.5 ± 1.2 mmol l^−1^), 1 showed no change and 1 showed a decline (Supplementary Table S1). Glucose levels upon capture ranged from 3.1 to 19.7 mmol l^−1^ (mean = 8.6 mmol l^−1^, *n* = 9). Following time on deck glucose levels ranged from 2.9 to 22.7 mmol l^−1^ (mean = 6.9 mmol l^−1^, *n* = 11). Of the seven sharks tested twice, only two showed increased glucose levels following time on deck and five showed a decline (mean decrease ± SD = 3.3 ± 6.9 mmol l^−1^) (Supplementary Table S1).

## Discussion

By combining biologging, temperature measurements immediately after capture and blood biochemistry, we show that capture significantly and rapidly (up to 0.8°C min^−1^) elevates the body temperature of sharks. Subcutaneous muscle temperature was elevated by as much as 2.7°C immediately after capture, and temperature increased deeper into the white muscle, but the magnitude of the temperature elevation above ambient was unrelated to shark size, hooking duration and blood lactate or glucose levels. Following release, T_b_ rapidly declined toward T_a_ during the first 30 mins for tiger sharks and 15 mins for blue sharks, and generally approximated T_a_ within 1 h after release. These results provide new insight into the stress physiology of capture in sharks and highlight the significant influence the fishing process has on shark body temperatures.

Although body temperature elevation has been acknowledged to be of importance when assessing the negative physiological impacts of catch-and-release fishing in the past, it has not been directly recorded under these conditions before. Only through these direct, fine-scale measurements were we able to show definitively that catch-and-release events caused an elevation of the body temperature of sharks. Acute body temperature elevations have been known to cause a number of physiological, ecological and behavioural consequences; in terms of physiology, rapid elevations of body temperature can speed up digestion rates (Carey *et al.,* 1984), increase the expression of heat shock proteins (Renshaw *et al.,* 2012) and affect biological rates, such as metabolism (Morley *et al.,* 2019), potentially contributing to a deficit in the sharks’ daily energy budget, as has been shown before (Bouyoucos *et al.,* 2017, 2018). Regarding behavioural changes, elevations of body temperature can result in distributional changes as sharks are known to adjust their position in the water column as a means to behaviourally thermoregulate (Sims *et al.,* 2006; Nakamura *et al.,* 2020; Watanabe *et al.,* 2021), or may expand their horizontal range if topographically limited (e.g. the tiger sharks in the Bahamas are depth limited and therefore may travel further offshore to seek out colder, deep waters) (Gallagher *et al.,* 2021). While the magnitude of the measured temperature increase deeper into the muscle (0.7°C–1.2°C on average) may not seem dramatic, muscles deeper into the body were higher than subcutaneous tissues for blue sharks, and the subcutaneous temperatures likely underestimate the true temperature elevation of those tissues. This is because we could not measure T_b_ of sharks prior to capture, so we calculated our estimates of T_b_ elevation based on sea surface temperatures at the point of capture and our biologging data, which showed shark T_b_ matches water temperature following sufficient equilibration time after release. Accordingly, our estimates of capture-induced heating of subcutaneous tissues are likely conservative because shark T_b_ may have been lower than surface temperatures if they had been inhabiting cooler (deeper) waters immediately prior to taking the hook, i.e. the true magnitude of heating could be higher than what we report. We do however acknowledge the limited sample size in our study; further collection of measurements of this kind from additional species and individuals could investigate any potential interindividual or interspecies variation. Future work could build on ours and other studies (Wosnick *et al.,* 2018, 2019) that explore how heat is distributed throughout the body of sharks and the associated physiological implications. It is also noteworthy that we report T_b_ to match T_a_ for these animals after they have recovered from capture (Fig. 1C and D), because few studies have equivocally shown this to be the case for large ectotherms that have significant thermal inertia (low temperature rate constants; Nakamura *et al.,* 2020).

The simultaneous elevation of body temperature and lactate concentration are undoubtedly due to higher metabolic rates during capture, which is partially through anaerobic pathways. Nevertheless, temperature elevation was not correlated with lactate concentration, and neither were correlated with hooking duration (if anything, all these relationships were slightly negative; Fig. 5). These results were somewhat unexpected, notwithstanding the known lag issues associated with using blood lactate as a direct proxy of the extent of cumulative anaerobic metabolism (blood lactate is chiefly a measure of anaerobic metabolism as it shifts from the white muscle to the blood when an animal switches from aerobic to anaerobic respiration during periods of increased energetic demands; Prohaska *et al.,* 2021), and that other studies have also shown hooking duration as a poor proxy of blood lactate levels (Shea *et al.,* 2022). Further studies with increased sample sizes would be beneficial to investigating this relationship further. Notwithstanding this, our data show that shark body temperature rapidly responds to capture but is not a proxy of blood lactate concentrations over the same time scales. Body temperature should therefore be treated as a new physiological proxy of exhaustion in captured sharks that reflects increased aerobic exercise as well as the mismatch between heat generated in the skeletal muscles and lost at the gills.

Capture-induced heating rates were far more rapid than what our biologged sharks exhibited naturally; some blue sharks heating almost eight times faster than did wild sharks swimming throughout thermally stratified waters. An important next step would be to determine the physiological implications of heating at these rates and magnitudes. There exists a rich literature on physiological responses to acute heating in the laboratory, which provides useful context. For example, Morley *et al.* (2019) compiled data on studies testing the upper temperature limits of marine, freshwater and terrestrial ectotherms under varying rates of warming. The maximum heating rates used in most experimental studies were ~1°C min^−1^, with most commonly used rates being much lower. These higher rates are often considered to be so fast as to not be ecologically meaningful (Payne *et al.,* 2016, 2021) whereas our sharks heated close to those maximum heating rates manipulated in the laboratory (0.8°C min^−1^). Heating rate has a well-recognised impact on an ectotherm’s thermal limit (Peck *et al.,* 2009; Kingsolver and Umbanhowar, 2018), so it could be instructive to determine physiological implications of this fast heating over the magnitude of temperature increases we documented. This could take the form of laboratory-based studies on physiological indicators of stress in sharks, which are undergoing thermal ramping, such as blood biochemistry, behavioural changes and response to stimuli. Observations from these controlled environments may aid our understanding of how this rapid heating is occurring and affecting the animals physiologically. Another avenue of research could be to explore behaviour post-release through biologging and investigate any relationship with ΔT. Increasing the number of individuals and species of varying lifestyles (e.g. sedentary, active predators) tagged with biologgers would greatly aid these studies.

Future research of this kind, and our own study, have numerous physiological and fisheries-based implications. Firstly, the findings of this study could be incorporated into future ecological models aimed at predicting mortality following catch-and-release angling, perhaps by incorporating a thermal threshold value. Moyes *et al.* (2006) constructed a model to predict the long-term survival of fish released following capture based on several blood markers (including lactate). This study noted that the inclusion of water temperature could have improved their model, with lower water temperatures likely resulting in reduced mortality post-release. We posit that this model could be further improved by the inclusion of body temperature in conjunction with water temperature, as we have shown that body temperature does not directly mimic water temperature in these ectothermic sharks for the first hour(s) after release. We are not aware of any other published studies that documented capture-induced changes in body temperature in large fishes, aside from a report on bluefin tuna captured in nets, which reported a ~2°C increase in body temperature (Addis *et al.,* 2009). Tunas, along with some other species such as lamnid sharks, have specialised physiology, which allows them to retain metabolically derived heat through vascular countercurrent heat exchangers, so could be expected to exhibit even greater temperature elevation during capture than ectothermic species. Many of those species are also of conservation concern, so it could be instructive to explore how catch-and-release fishing might impact the welfare of these animals via T_b_ elevations.

In summary, we show that catch-and-release angling causes a measurable and rapid increase in the body temperature of sharks. With fast temperature elevations of up to 2.7°C, and possibly greater, it might be important for future research to explore any physiological impacts that this heating might cause, to better manage catch-and-release programs. Welfare outcomes of different handling protocols have been examined in other species (Danylchuk *et al.,* 2007; Brownscombe *et al.,* 2017; Raoult *et al.,* 2019) and exploring implications of factors such as removing sharks from the water (versus leaving them submerged) or catching them near the upper limit of their thermal niche could be helpful for this group of animals.

## Funding

This work was supported by Science Foundation Ireland [grant number 18/SIRG/5549 to N. P.]; and the Irish Research Council [grant number IRCLA/2017/186 to A. J.].

## Data availability

The data underlying this article are available in the article and in its online supplementary material.

## Conflicts of Interest

We know of no conflicts of interest associated with this publication and that any research in the paper not carried out by the authors is fully acknowledged in the manuscript. All sources of funding are acknowledged in the manuscript, and authors have declared any direct financial benefits that could result from publication.

## Supplementary Material

Web_Material_coac065
