# Supplementary material for: Capture heats up sharks
Source: Conserv Physiol. 2022 Sep 28;10(1):coac065. doi: 10.1093/conphys/coac065 (PMC9517936; doi:10.1093/conphys/coac065)
Supplement: Web_Material_coac065 [file web_material_coac065.docx]

Supplementary Information

Capture heats up sharks.

Table 1: Biometrics and measurements taken of all animals included in this study; for restraint method, OD = on deck, & SB = secured alongside the boat; TL = total length (cm), FL = fork length (cm), BM = estimated body mass (kg), HT = hooking time (mins), T_b_ = body temperature upon capture (°C), T_a_ = water temperature upon capture (°C), Lac1 = lactate upon capture (mmol/L), Lac2 = lactate immediately prior to release (mmol/L), Gluc1 = glucose upon capture (mmol/L), Gluc2 = glucose immediately prior to release (mmol/L).

| **Date** | **Animal ID** | **Species** | **Sex** | **Fishing method** | **Restraint method** | **Biologging package** | **TL (cm)** | **FL (cm)** | **BM (kg)** | **HT (mins)** | **T_b_ (°C)** | **T_a_ (°C)** | **Lac1** | **Lac2** | **Gluc1** | **Gluc2** |
| --- | --- | --- | --- | --- | --- | --- | --- | --- | --- | --- | --- | --- | --- | --- | --- | --- |
| 02.05.2019 | T1 | Tiger shark | Female | Drum line | SB | Yes | 298 | 245 | 155.67 | NA | 27.7 | 26.8 | NA | NA | NA | NA |
| 04.05.2019 | T2 | Tiger shark | Female | Drum line | SB | Yes | 311 | 266 | 203.5 | NA | NA | NA | NA | NA | NA | NA |
| 06.05.2019 | T3 | Tiger shark | Female | Drum line | SB | Yes | 316 | 264 | 198.59 | NA | 28.1 | 27.6 | NA | NA | NA | NA |
| 07.05.2019 | T4 | Tiger shark | Female | Drum line | SB | Yes | 323 | 267 | 206.04 | NA | 28.8 | 28.2 | NA | NA | NA | NA |
| 27.09.2019 | BSCC | Blue shark | Male | Angling | OD | Yes | 213 | 170 | 30.70 | 13 | 18.5 | 17.8 | NA | NA | NA | NA |
| 17.07.2021 | BS09 | Blue shark | Female | Angling | OD | Yes | 195 | 160 | 25.39 | 6 | 19.1 | 18.5 | NA | 9.0 | NA | 22.7 |
| 18.08.2021 | BS21 | Blue shark | Female | Angling | OD | No | 162 | 133 | 14.24 | 4 | 15.9 | 13.9 | 2.0 | 4.4 | NA | NA |
| 18.08.2021 | BS22 | Blue shark | Female | Angling | OD | No | 196 | 161 | 25.90 | 5 | 16.6 | 15.3 | 1.9 | 3.4 | 4.4 | 4.1 |
| 18.08.2021 | BS23 | Blue shark | Female | Angling | OD | No | 211 | 168 | 29.59 | 4 | 16.2 | 15.4 | 9.0 | 5.7 | NA | 2.9 |
| 18.08.2021 | BS24 | Blue shark | Female | Angling | OD | No | 193 | 159 | 24.90 | 4 | 17.5 | 14.8 | 7.5 | 7.5 | NA | 3.6 |
| 18.08.2021 | BS25 | Blue shark | Female | Angling | OD | No | 161 | 132 | 13.90 | 1.4 | 15.9 | 14.8 | 1.2 | NA | 18.9 | NA |
| 18.08.2021 | BS26 | Blue shark | Female | Angling | OD | No | 193 | 159 | 24.90 | 3.9 | 16.9 | 14.8 | 1.5 | 3.0 | 19.7 | 4.1 |
| 19.08.2021 | BS27 | Blue shark | Female | Angling | OD | No | 212 | 176 | 34.23 | 4 | 16.7 | 16.0 | 1.5 | 2.9 | NA | 4.1 |
| 19.08.2021 | BS28 | Blue shark | Male | Angling | OD | No | 161 | 132 | 13.90 | 4 | 17.4 | 16.5 | 3.5 | 7.1 | 15.6 | 19.5 |
| 15.09.2021 | BS30 | Blue shark | Female | Angling | OD | No | 159 | 132 | 13.90 | 2.25 | 18.6 | 17.4 | 3.3 | 4.4 | 4.5 | 4.3 |
| 05.11.21 | BS32 | Blue shark | Female | Angling | OD | No | 204 | 169 | 30.14 | 6.3 | 14.3 | 13.3 | 1.4 | 5.7 | 3.1 | 3.4 |
| 05.11.21 | BS33 | Blue shark | Female | Angling | OD | No | 232 | 191 | 44.22 | 9.8 | 13.6 | 13.0 | 1.8 | 5.8 | 3.6 | 3.4 |
| 05.11.21 | BS34 | Blue shark | Female | Angling | OD | No | 191 | 173 | 32.43 | 11.2 | 14.0 | 12.9 | 2.1 | NA | 3.7 | NA |
| 05.11.21 | BS35 | Blue shark | Female | Angling | OD | No | 237 | 194 | 46.43 | 7.3 | 14.5 | 12.9 | 3.1 | 5.6 | 3.7 | 3.4 |

Table 2: Fitting of regression models where “deltaT” is T_b_ – T_a_ (i.e. ΔT) upon capture; “Lact” is lactate concentration upon capture; “Gluc” is glucose concentration upon capture; “log_bm” is the log of body mass (kg); and “hooking_time” is duration of time from moment of hooking to moment of landing on deck (mins).

| Model | R^2^ | AIC |
| --- | --- | --- |
| deltaT ~ Lact + Gluc + log_bm + hooking_time | 0.4452 | 16.51698 |
| deltaT ~ Lact + Gluc + log_bm*hooking_time + hooking_time | 0.584 | 15.92566 |
| deltaT ~ log_bm + hooking_time | 0.07667 | 32.80171 |
| deltaT ~ Lact + hooking_time | 0.1852 | 28.98296 |
